# Supplementary material for: An Interaction Network of RNA-Binding Proteins Involved in Drosophila Oogenesis
Source: Mol Cell Proteomics. 2020 Nov 25;19(9):1485–502. doi: 10.1074/mcp.RA119.001912 (PMC8143644; doi:10.1074/mcp.RA119.001912)

Fig. 1
IP: anti-GFP

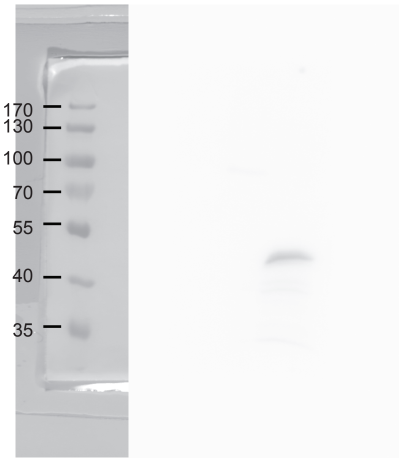

Control (tag)

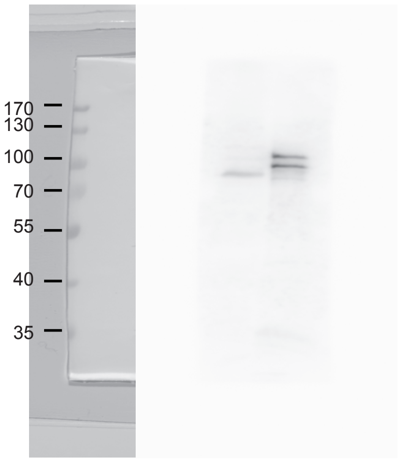

eIF4AIII

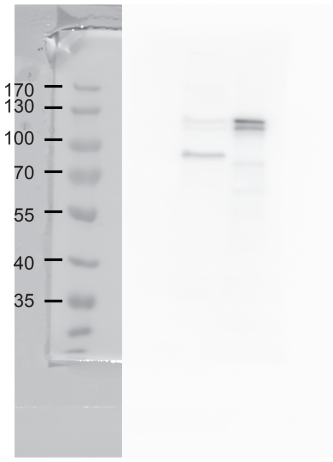

Glo

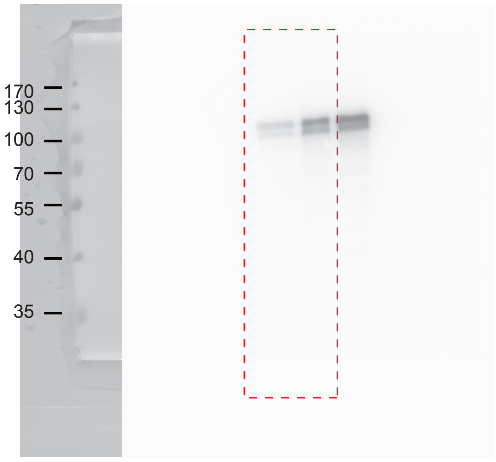

Hrp48

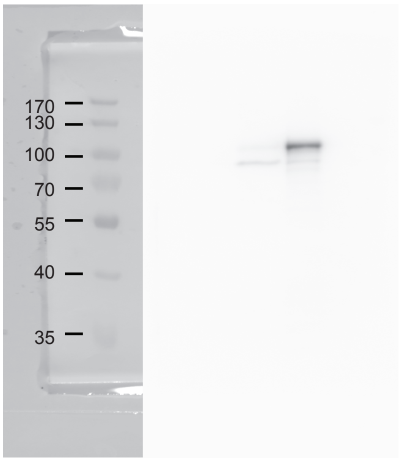

Nos

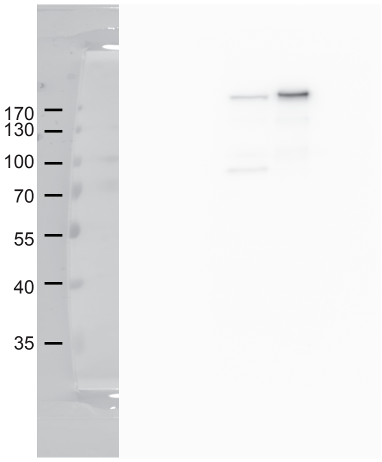

Stau

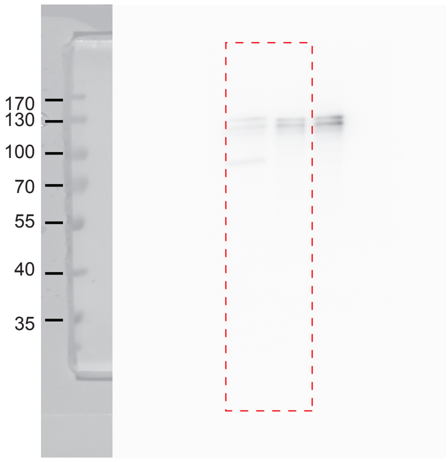

Vas

Fig. 7 a) **Bait: GFP-eIF4AIII**
IP: anti-GFP

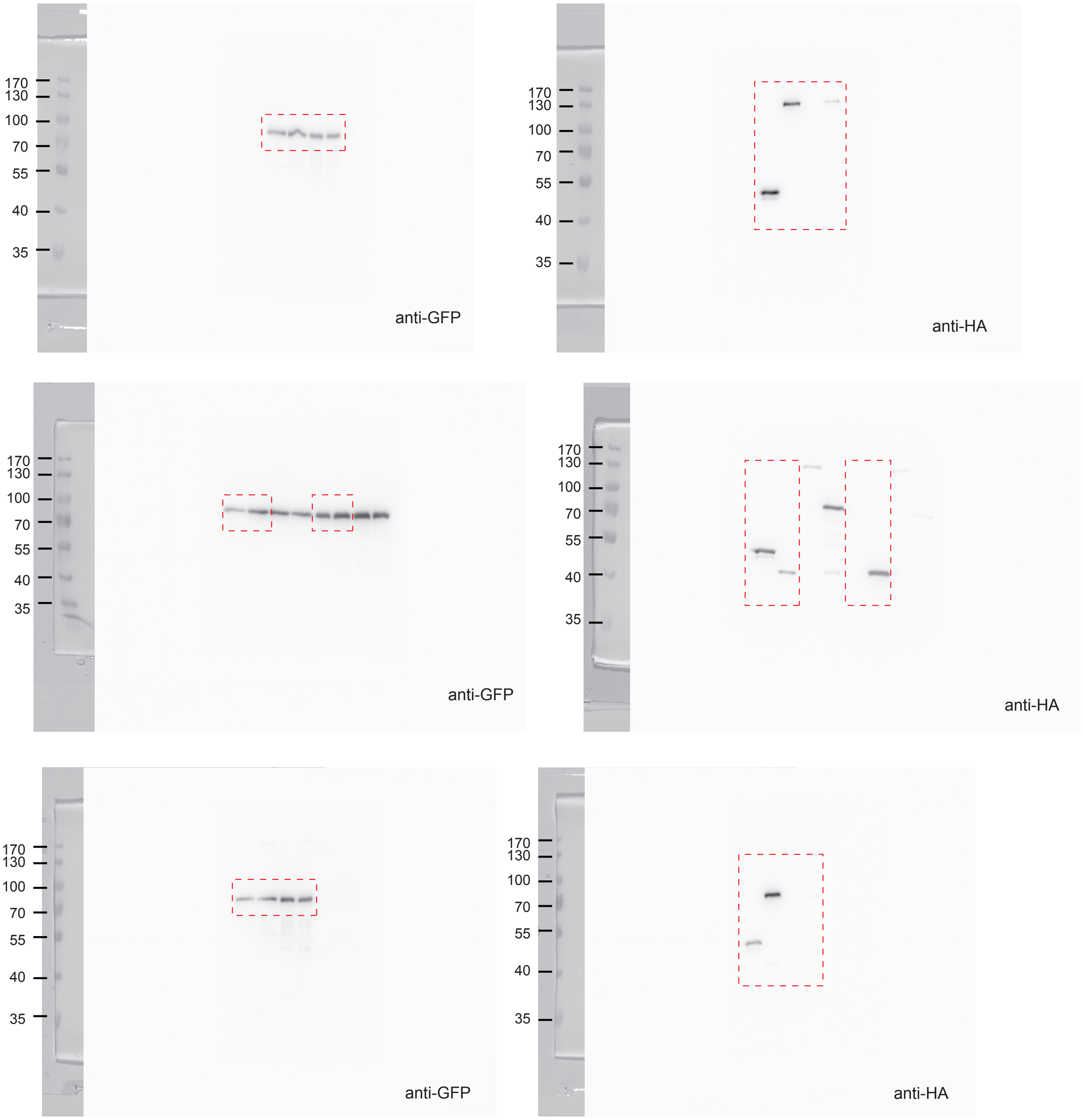

b) **Bait: GFP-Glo**
IP: anti-GFP

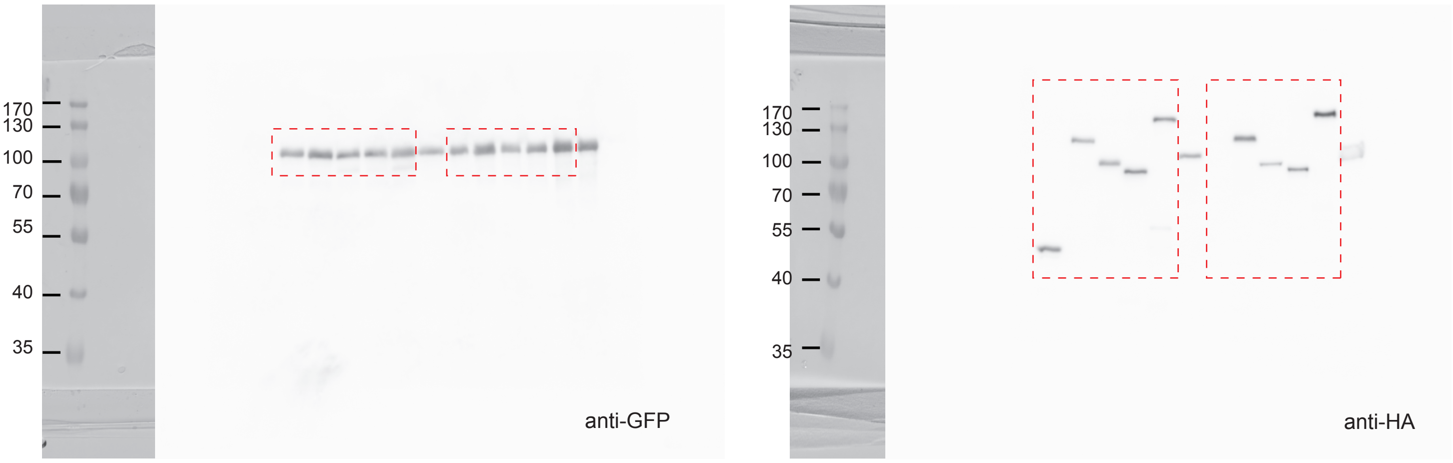

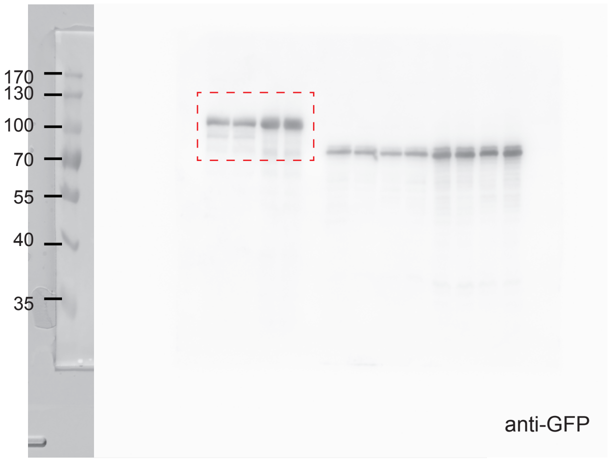

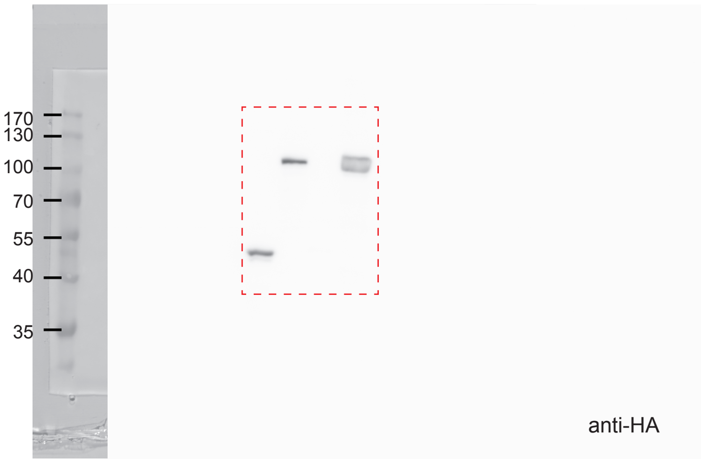

c) Bait: GFP-Nos
IP: anti-GFP

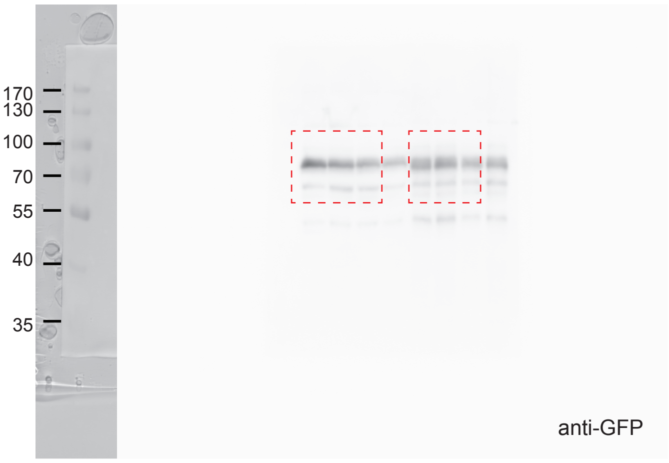

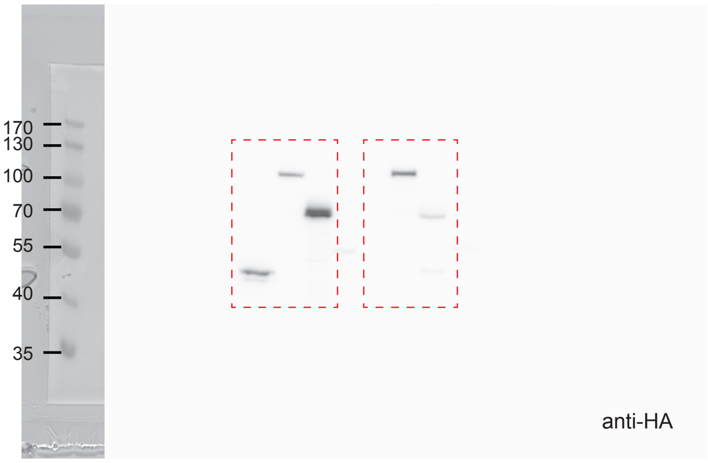

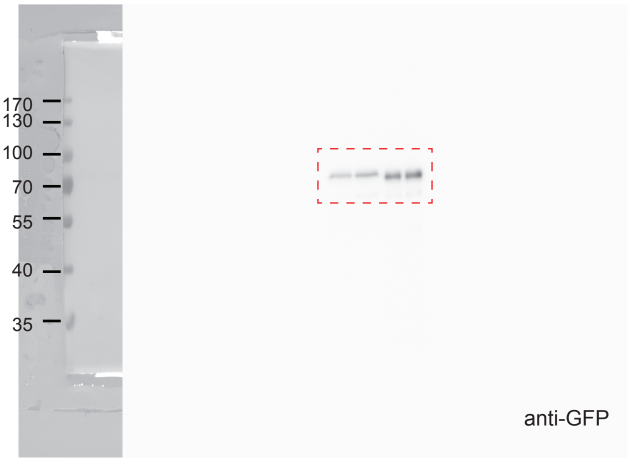

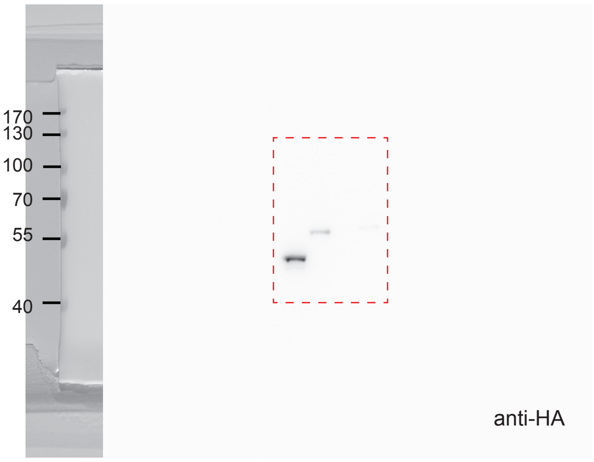

d) Bait: GFP-Vas
IP: anti-GFP

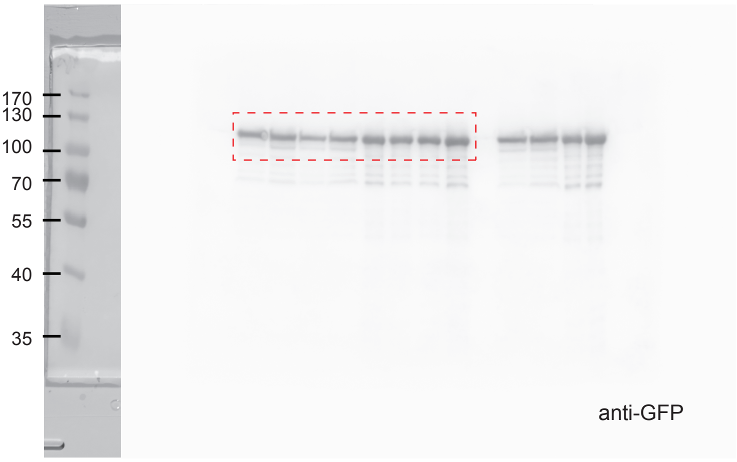

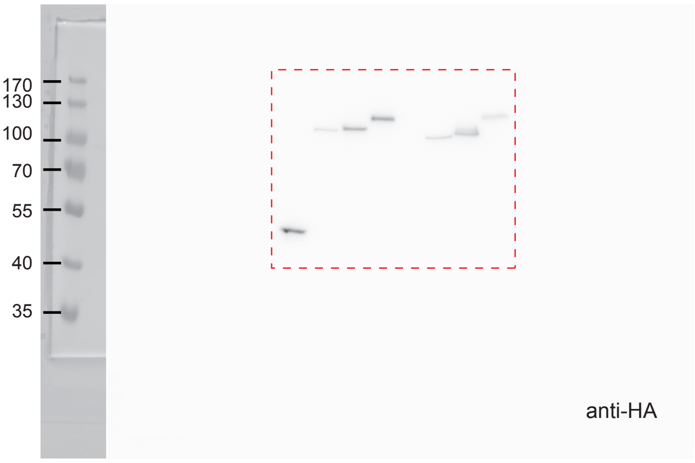

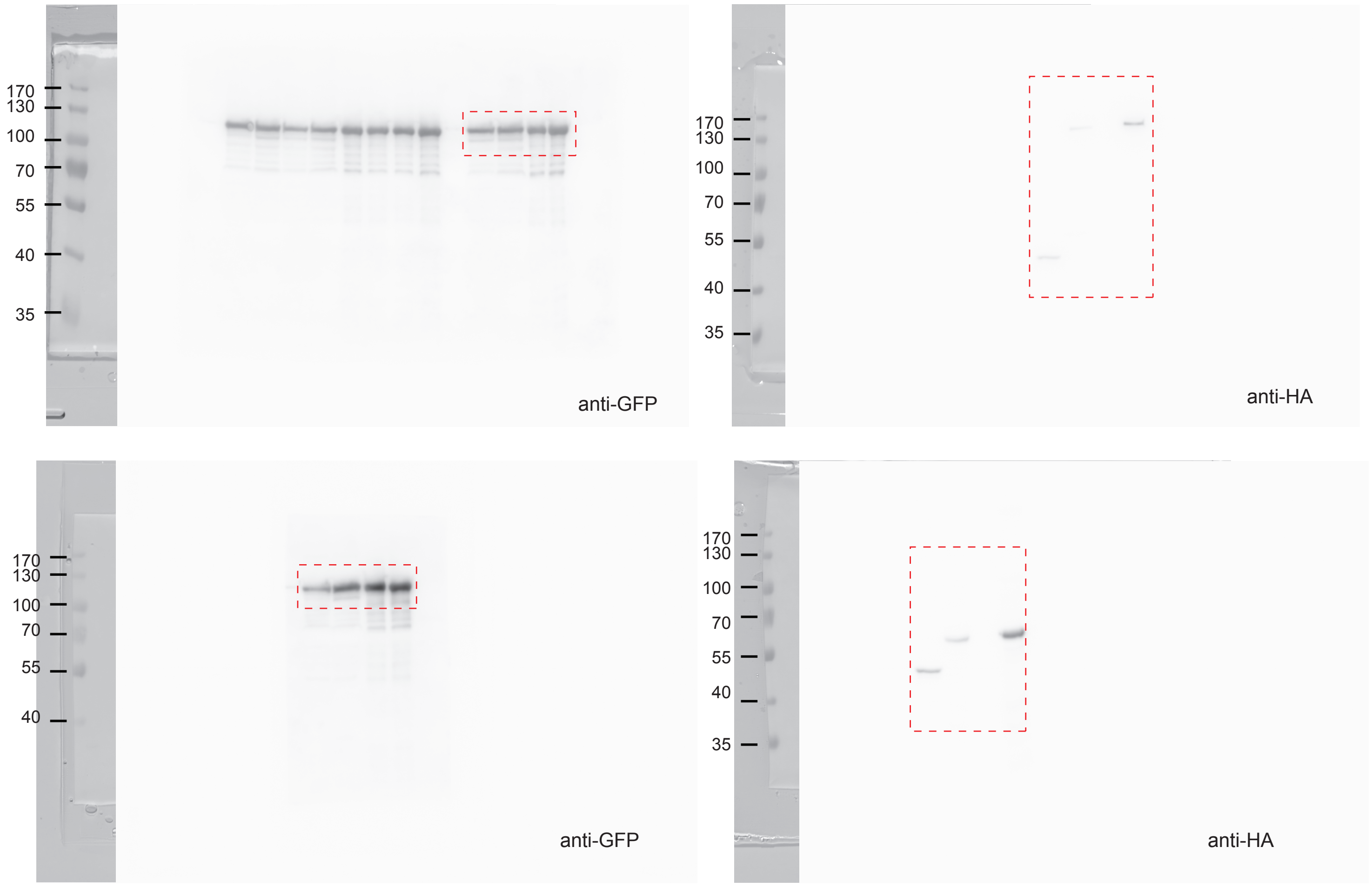

e) Bait: GFP-Hrp48
IP: anti-GFP

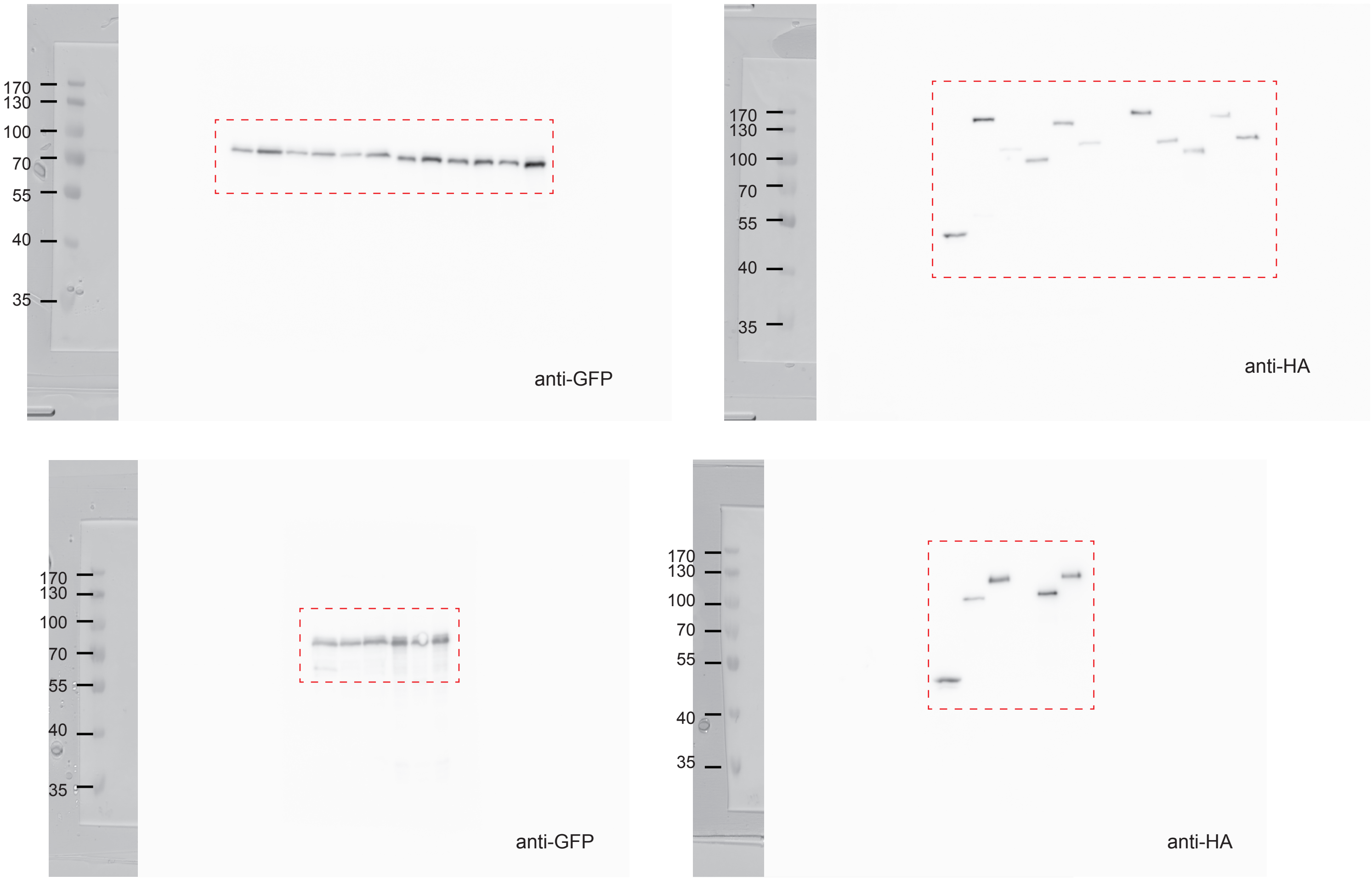

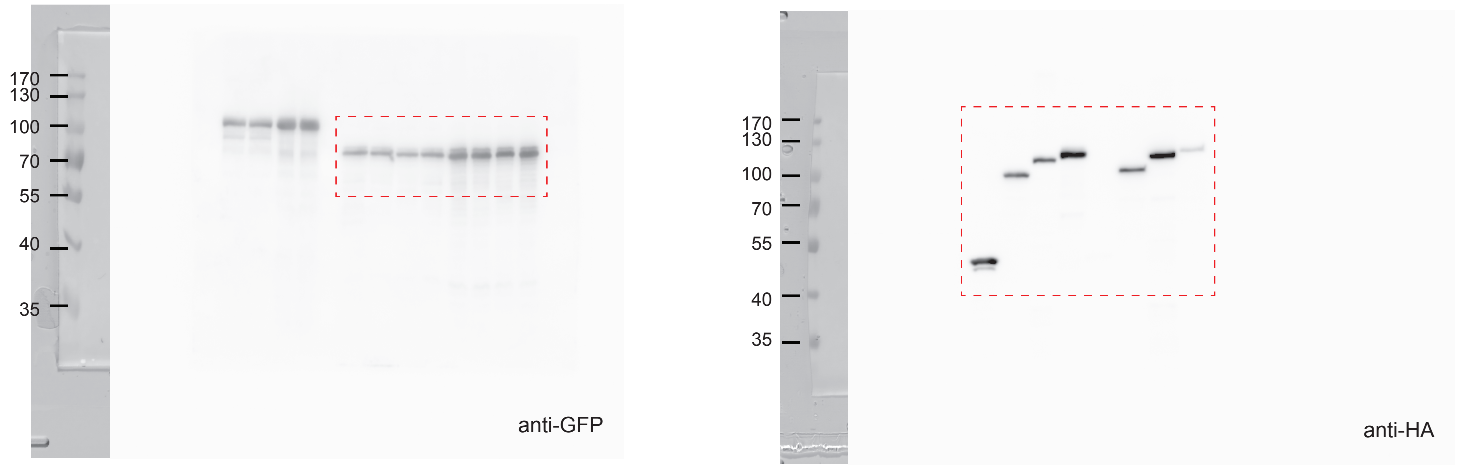

f) Bait: HA-Flag-Stau
IP: anti-Flag

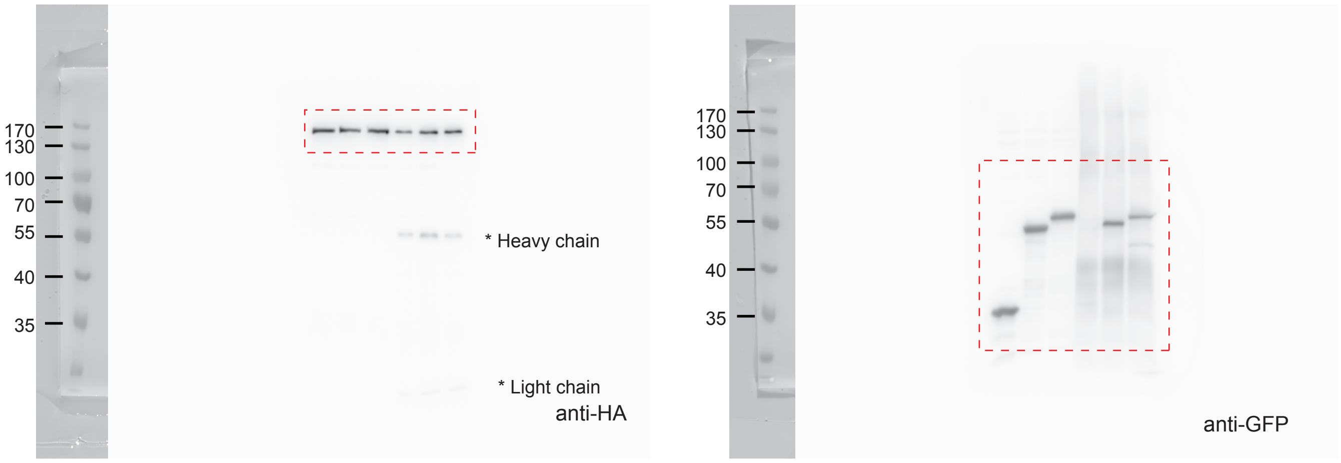

g) Bait: GFP-Stau
IP: anti-GFP

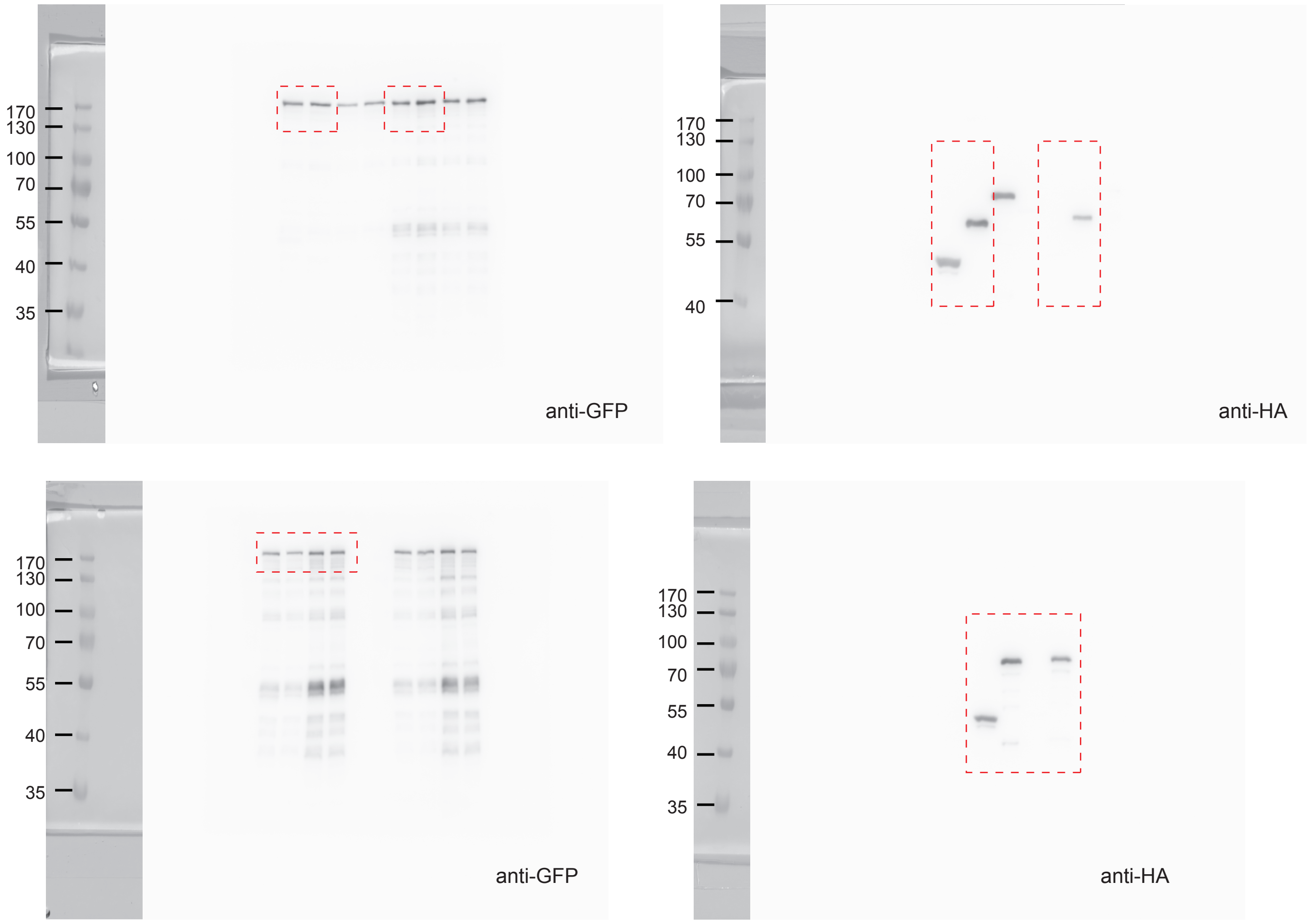

h) **Bait: HA-Flag-Glo**
IP: anti-Flag

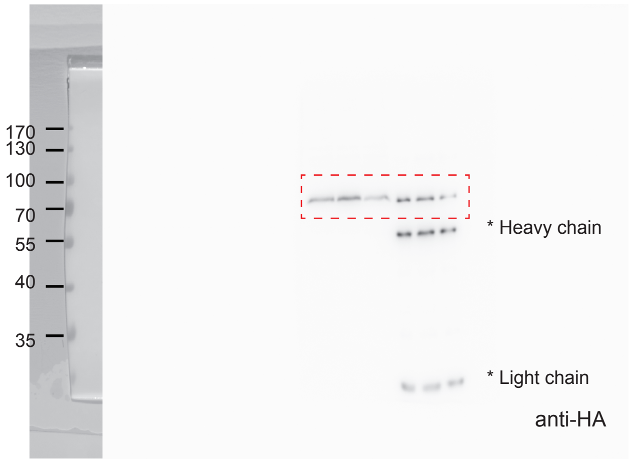

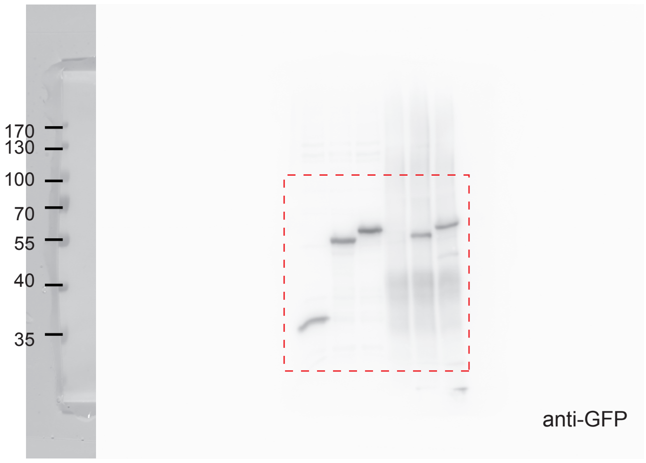

i) **Bait: GFP-Glo**
IP: anti-GFP

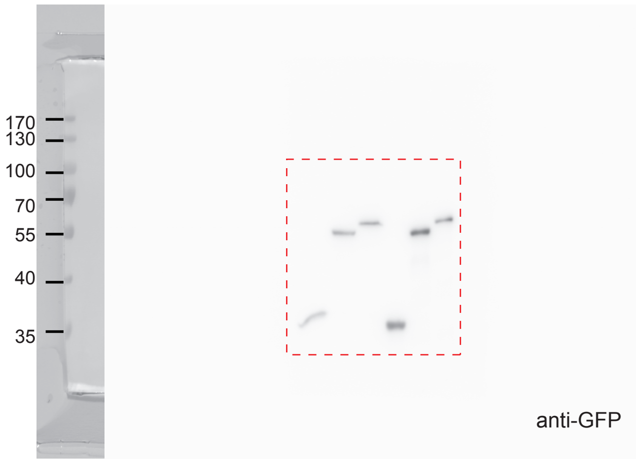

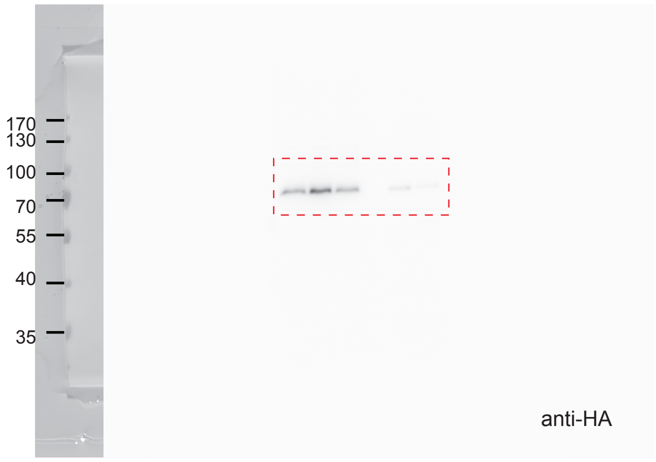

Supplement: Supplementary file 1 [file mmc1.zip › mmc1/157750_1_supp_536521_qxy2q7.pdf]
