# Supplementary material for: An Interaction Network of RNA-Binding Proteins Involved in Drosophila Oogenesis
Source: Mol Cell Proteomics. 2020 Nov 25;19(9):1485–502. doi: 10.1074/mcp.RA119.001912 (PMC8143644; doi:10.1074/mcp.RA119.001912)
Supplement: Supplementary file 1 [file mmc1.zip › mmc1/157750_1_supp_539316_qb3gl8.pdf]

An interaction network of RNA-binding proteins involved in *Drosophila* oogenesis

Prashali Bansal, Johannes Madlung, Kristina Schaaf, Boris Macek, Fulvia Bono*

Supplemental Data:

- Supplemental Methods
- Supplemental Tables
- References
- Supplemental Figures
- Supplementary files: Supplemental data S1: GO analysis
  Supplemental data S2: Statistics Table for label-free MS
  Supplemental data S3: Cytoscape network (related to Fig. S4)
  Supplemental data S4: Gephi file (related to Fig. 4a)
  Supplemental data S5: Interactions from literature
  Supplemental data S6: Raw immunoblots
  Supplemental data S7: Single peptide identifications, dimethyl MS

Supplementary Methods

Genetic rescue of Flyfos lines

Fly lines used for rescue experiments were purchased from Bloomington *Drosophila* Stock Center (BDSC 2781; 7626; 3285; 7662; 5011; 6058; 57693; 7965; 8847; 10375). Fly lines expressing *stau* mutant *stau[D3] and stau[R9]* have been previously described (1). To check if the tagged proteins are functional, transgenes were tested against a deficiency in *trans* to a mutant allele, or two mutant alleles as in the case of *stau*, for rescue of the respective phenotypes. For *nos*, the transgene was recombined with a *nos* deficiency, since the gene is located on the same chromosome as the insertion. The resulting *trans*-heterozygotes were checked for embryonic lethality or female sterility, to assess the functionality of the transgenes. For maternal effect genes (phenotype of the individual is dependent on the genotype of the mother), *trans*-heterozygotes

were further crossed with wild-type males and the progenies were examined for their ability to rescue the phenotype. dsRed was used as a selection marker for all the transgenes in every generation.

Immunofluorescence and Microscopy

To visualize the GFP-tagged proteins *in vivo*, ovaries from well-fed flies were dissected in PBS at RT and fixed in 4% PFA (in PBS) for 4 min. After washing in PBS, samples were mounted directly in *Fluoromount-G*™ (Southern Biotech). Antibody staining was done as previously described (2) using anti-GFP (1:1000, Thermo Fisher Scientific A11122), except that primary antibody incubation was done for 3 days at RT. Images were acquired on a FluoView1200 laser scanning confocal microscope (Olympus), with an UPlanSApo 40.0X air objective (NA 0.95) and processed using FIJI imaging software (3).

Supplementary Tables

Table S1: GenBank Accession numbers of genes cloned for mammalian cell expression

**Genes subcloned from cDNA**	**GenBank Accession**
Casein kinase II alpha (CkIIalpha)	AAN11415.1
Armitage (Armi)	AAT12000.1
CG13090 (Uba4)	AGB92792.1
CG5726	AAF57731.1
CG7185	AAF50445.2
CG9684	AAF54236.2
Coilin (isoform D) (Coil)	AAX52725.1
Discs overgrown (Dco)	QCD25211.1
Disco interacting protein 1 (isoform D) (Dip1)	AAN09007.1
DNA replication-related element factor (Dref)	BAA24827.1
Gustavus (Gus)	AAF57346.2
Heat shock protein 60A (Hsp60)	AAF47999.1

Loquacious (isoform PB) (Loqs)	AAF53295.2
Nucampholin (Ncm)	AAF53667.3
NudC	AAF49407.2
Half pint (Hfp)	AAF47501.1
Nucleophosmin (Nph)	AAF56987.1
X16 splicing factor (X16)	AAF52454.1
SR family splicing factor 35 (SC35)	AAF43415.1
eIF4AIII	AAF54221.1
Heterogeneous nuclear ribonucleoprotein at 27C (Hrp48)	AAF52456.1
Nanos (isoform B) (Nos)	AGB96103.1
Staufen (Stau)	AAF57752.1
Fragile X mental retardation protein 1 (Fmr1) (Isoform F)	ACZ94877.1
Cup	AAF52418.3
Decapping protein 1 (DCP1)	AAF47089.1
eIF4E (isoform C)	AAF50281.1
oo18 RNA-binding protein (Orb)	AAF56120.1
Hephaestus (Heph) (Isoform Z)	AHN57629.1
Ovarian tumor (Otu) (Isoform A)	AAF46384.1
Squid (isoform C) (Sqd)	AAN13570.1
Up-frameshift 1 (Upf1)	AAF48115.2
Belle (Bel)	AAF54262.1
Exuperantia (Exu)	AAF57429.1
Trailer hitch (Tral) (Isoform A)	AAF49905.3
Barentz (Btz)	AAF56741.1
Splicing factor 2 (SF2)	AAF43413.1
Small ribonucleoprotein particle protein SmB (SmB)	ADV37030.1
mitochondrial single stranded DNA-binding protein (mtSSB)	AAF55287.2
Lost	AAF52130.1
Maternal expression at 31B (Me31B)	AAF52881.2
Oskar (isoform short)	AAS65129.1
Bruno (isoform A)	AAN10812.1
Glorund (Glo)	AAF54704.1
Vasa (Vas)	AAF53438.1
**Genes subcloned from DGRC clones**	**Clone number**
CG10077	BO10625
Maheshvara (Mahe)	BO11418
Bicaudal C (BicC)	BS24007

Table S2: Summary of samples used for MS analysis

	No. of dissected flies (Label-free MS)			No. of dissected flies (Dimethyl labeling MS)	
	**Rep. I**	**Rep. II**	**Rep. III**	**Rep. I**	**Rep. II**
GFP	320	320	320	400	400
eIF4AIII-GFP	200	400	400	-	-
Glo-GFP	200	200	200	-	-
Hrp48-GFP	80	80	80	80	80
Nos-GFP	200	400	400	-	-
Stau-GFP	200	400	400	-	-
Vas-GFP	80	80	80	80	80

References

1. Lazzaretti, D., Bandholz-Cajamarca, L., Emmerich, C., Schaaf, K., Basquin, C., Irion, U., and Bono, F. (2018) The crystal structure of Staufen1 in complex with a physiological RNA sheds light on substrate selectivity. *Life Sci. Alliance* 1, e201800187

2. Palacios, I. M., and St Johnston, D. (2002) Kinesin light chain-independent function of the Kinesin heavy chain in cytoplasmic streaming and posterior localisation in the Drosophila oocyte. *Development* 129, 5473–5485

3. Schindelin, J., Arganda-Carreras, I., Frise, E., Kaynig, V., Longair, M., Pietzsch, T., Preibisch, S., Rueden, C., Saalfeld, S., Schmid, B., Tinevez, J.-Y., White, D. J., Hartenstein, V., Eliceiri, K., Tomancak, P., and Cardona, A. (2012) Fiji: an open-source platform for biological-image analysis. *Nat. Methods* 9, 676–682

Fig. S1

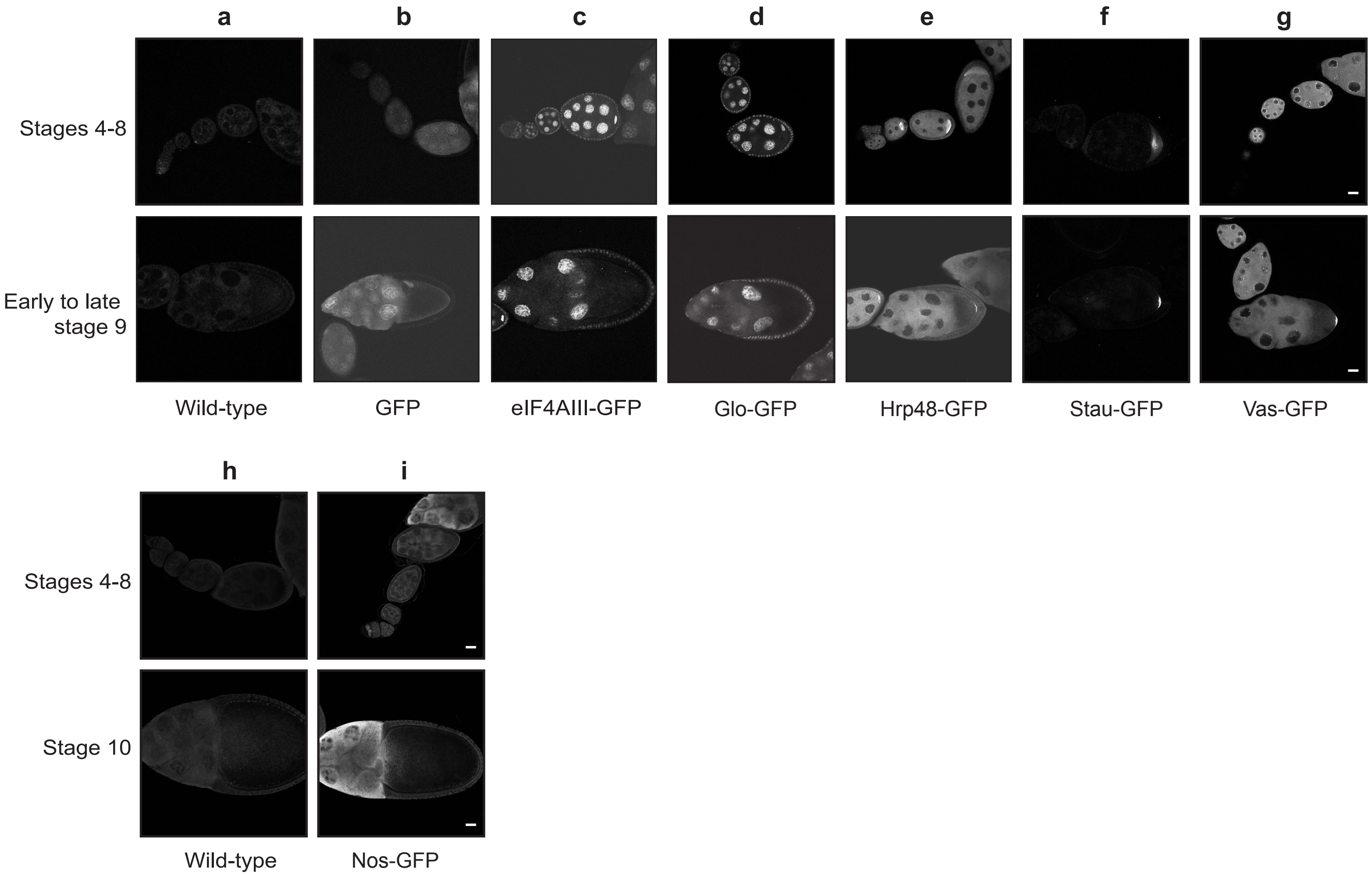

j

Gene	Chromosome	Transgene insertion on chromosome	Genotype of the mutants assayed for rescue	Rescue	Rescue assay
eIF4AIII	3	2	eIF4AIII-GFP/+ ; Df(3R)Exel614/CG7483[19]	No	Lethality not rescued
glo	3	2	Glo-GFP/+ ; Df(3R)Exel7310/glo[162x]	Yes	Lethality rescued to viable adults
hrp48	2	3	Df(2L)BSC108/hrp48[k02814] ; Hrp48-GFP/+	No	Lethality not rescued
nos	3	3	Df(3R)Exel6183 Nos-GFP/nos[L7]	Yes	Maternal effect. Lethality rescued to viable adults. Abdominal patterning defects observed in adults
stau	2	3	stau[D3]/stau[R9] ; Stau-GFP/+	No	Maternal Effect. Lethality not rescued
vas	2	3	Df(2L)A72/vas[RJ36] ; Vas-GFP/+	Yes	Maternal effect. Sterility rescued to fertile adults

Fig S1. Characterization of transgenic fly lines expressing GFP-tagged RBPs by analyzing localization patterns in the egg chambers and genetic rescue of the RBP fusions

(a-g) Images showing localization of the GFP-tagged proteins, in stages 4-9 egg chambers. Wild-type flies and flies expressing GFP serve as negative and positive controls, respectively. (h,i) Nos-GFP expressing ovaries immunostained for GFP show a uniform expression in the nurse cells of stage 10 egg chambers. Wild-type flies serve as a negative control. In panels a and b, GFP signal is shown. Anterior is to the left and posterior is to the right. Scale bar, 20 μm. (j) Table summarizing the genetic rescue of the mutant phenotypes with the transgenes. Respective genotypes of the mutants examined for the rescue assay to assess the functionality of the transgenes *in vivo*, are indicated. See Supplementray methods for details.

Fig. S2

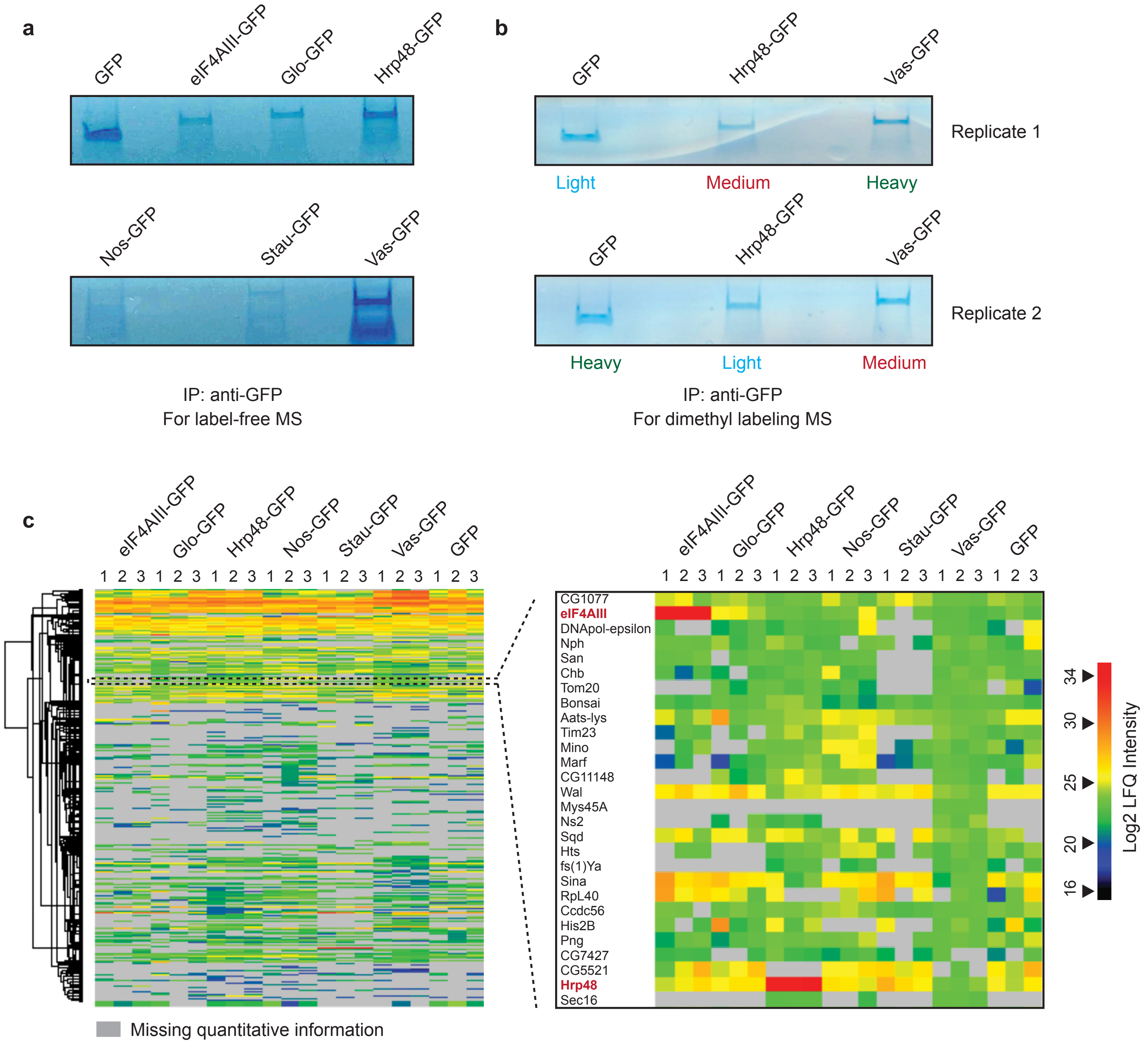

Fig S2. Baits and the interacting proteins were reproducibly enriched among replicates

Shown are the representative SDS-PAGE gels used for label-free (a) and dimethyl labeling (b) MS analysis. IPs were performed as described in the text and bands were visualized by Coomassie staining. IP from transgenic line expressing GFP served as a control. All baits could be purified successfully. Given the sensitivity of the assay, weak bands such as those of Nos-GFP and Stau-GFP were sufficient for the analysis. Please note that the samples were run for only about 2 cm into the gel, to minimize sample loss in the subsequent steps. (c) Heat map of the logarithmized (Log2) LFQ intensities of all proteins quantified in each sample, as analyzed by label-free MS. Hierarchical row clustering was performed on data without imputation. The zoomed area shows the consistent high enrichment of eIF4AIII-GFP and Hrp48-GFP (highlighted in red) in the three replicates. Missing quantitative information is highlighted in grey.

Fig. S3

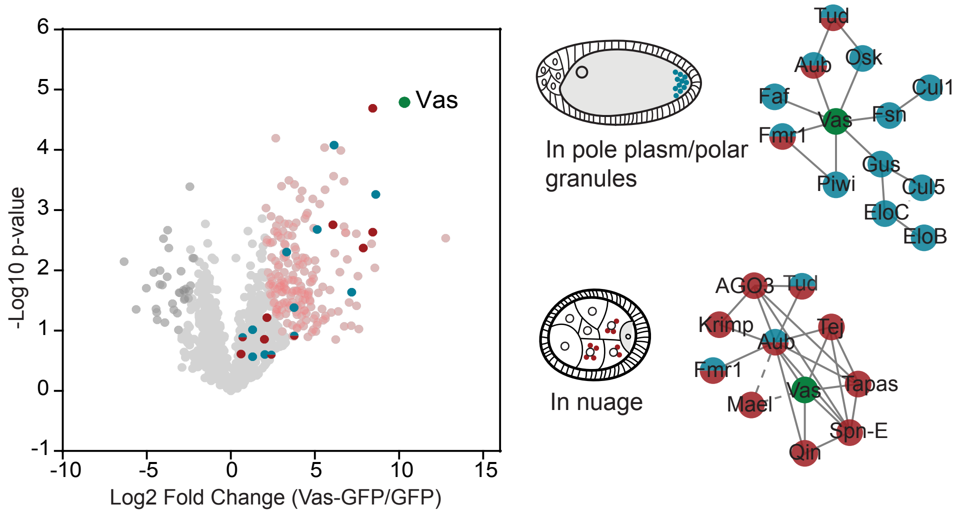

Fig S3. Vas-associated protein complexes functioning in polar granules and nuage were successfully co-purified with the transgene.

Scatter plot indicating components of functionally distinct pathways identified to be associated with Vas-GFP, in the label-free MS data. Each identified protein is represented as a dot in light grey; the bait is highlighted in green; significantly enriched proteins are highlighted in pink; proteins that associate in the nuage particles are highlighted in magenta; proteins involved in pole plasm assembly are highlighted in cyan; background binders are highlighted in dark grey. On the right, the interactions between the various components involved in the respective pathways are shown, as reported in literature. Solid line represents physical interactions; dotted line represents co-localization.

Fig. S4

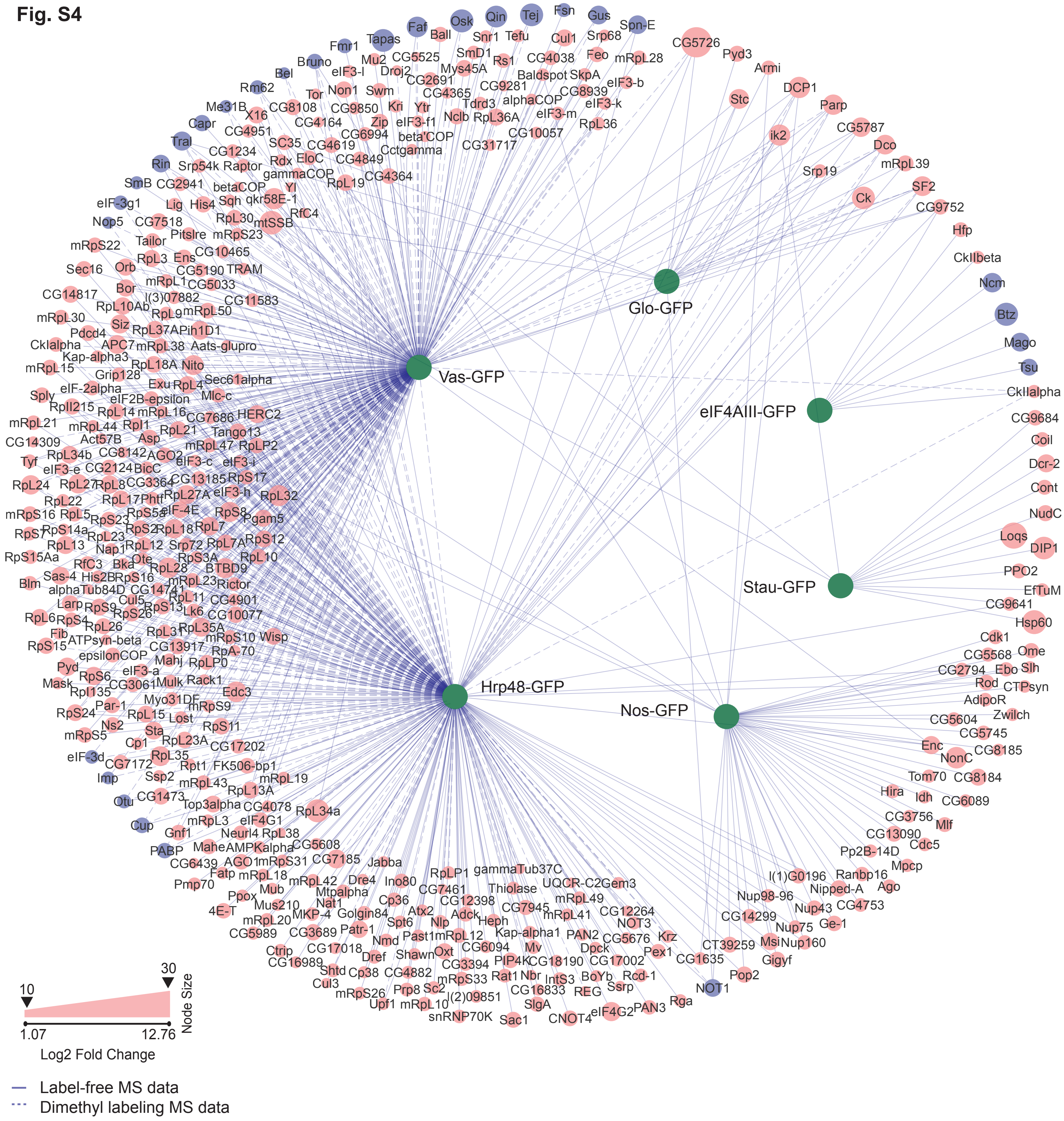

Fig S4. Global interactome unveils many shared interactants among selected RBPs

Shown is the interaction network of all the significantly enriched proteins, identified to be associated with each bait in the MS analysis. For Hrp48 and Vas, proteins from both label-free and labeled MS analyses were considered. Green nodes represent the baits; pink nodes represent the interactants; blue nodes represent the known interactants. Connecting edges highlight the interactions: solid edges represent the interactions found in label-free MS; dotted edges represent the interactions found in labeled MS. The size of the node (except for baits) indicate the fold change (Log2) over control.

Fig. S5

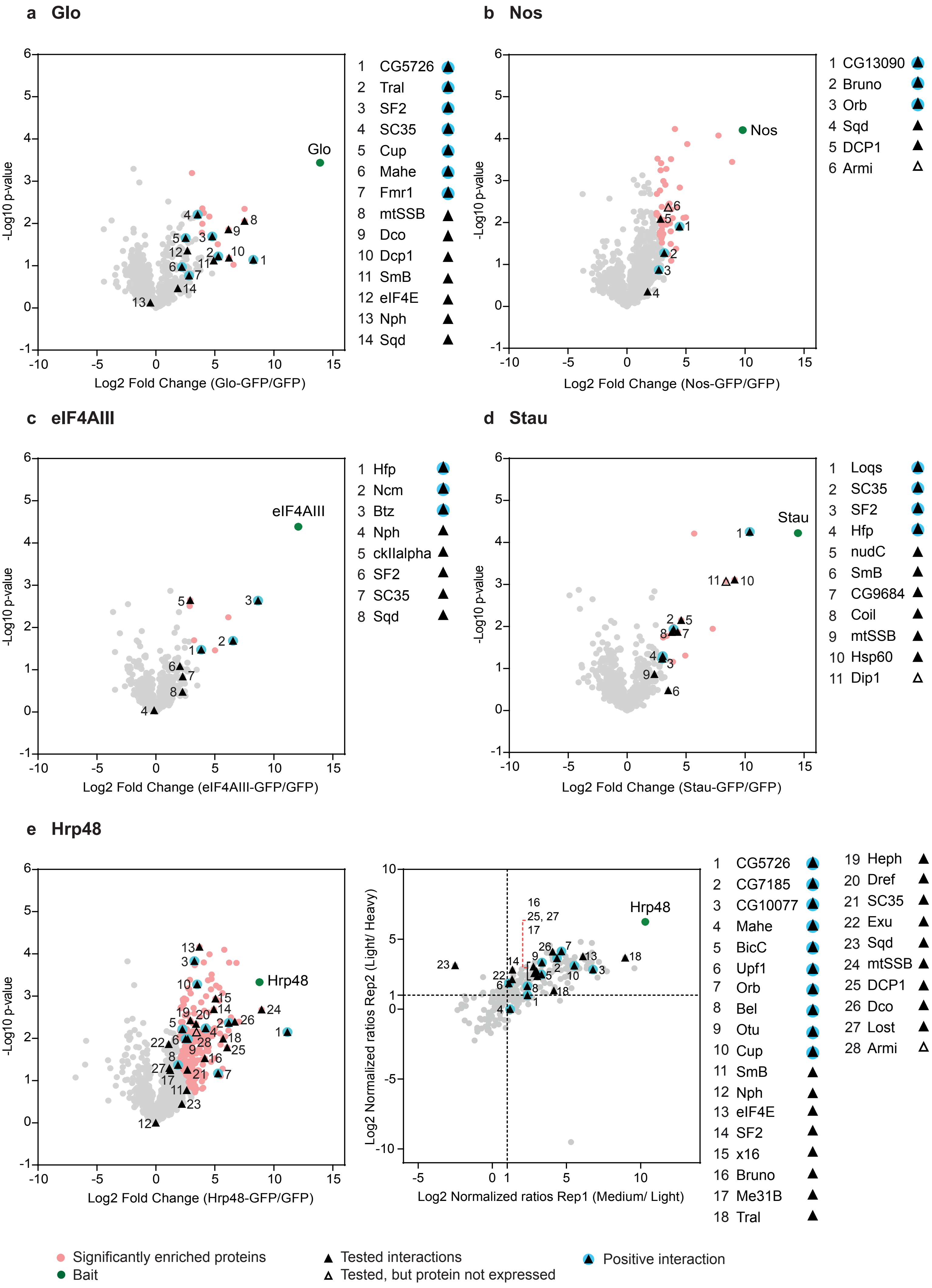
a Glo
Glo
-Log10 p-value
Log2 Fold Change (Glo-GFP/GFP)
1 CG5726
2 Tral
3 SF2
4 SC35
5 Cup
6 Mahe
7 Fmr1
8 mtSSB
9 Dco
10 Dcp1
11 SmB
12 eIF4E
13 Nph
14 Sqd
b Nos
Nos
-Log10 p-value
Log2 Fold Change (Nos-GFP/GFP)
1 CG13090
2 Bruno
3 Orb
4 Sqd
5 DCP1
6 Armi
c eIF4AIII
eIF4AIII
-Log10 p-value
Log2 Fold Change (eIF4AIII-GFP/GFP)
1 Hfp
2 Ncm
3 Btz
4 Nph
5 ckIIalpha
6 SF2
7 SC35
8 Sqd
d Stau
Stau
-Log10 p-value
Log2 Fold Change (Stau-GFP/GFP)
1 Loqs
2 SC35
3 SF2
4 Hfp
5 nudC
6 SmB
7 CG9684
8 Coil
9 mtSSB
10 Hsp60
11 Dip1
e Hrp48
Hrp48
-Log10 p-value
Log2 Fold Change (Hrp48-GFP/GFP)
Log2 Normalized ratios Rep2 (Light/ Heavy)
Log2 Normalized ratios Rep1 (Medium/ Light)
16
25, 27
17
1 CG5726
2 CG7185
3 CG10077
4 Mahe
5 BicC
6 Upf1
7 Orb
8 Bel
9 Otu
10 Cup
11 SmB
12 Nph
13 eIF4E
14 SF2
15 x16
16 Bruno
17 Me31B
18 Tral
19 Heph
20 Dref
21 SC35
22 Exu
23 Sqd
24 mtSSB
25 DCP1
26 Dco
27 Lost
28 Armi
Significantly enriched proteins
Bait
Tested interactions
Tested, but protein not expressed
Positive interaction

f Vas

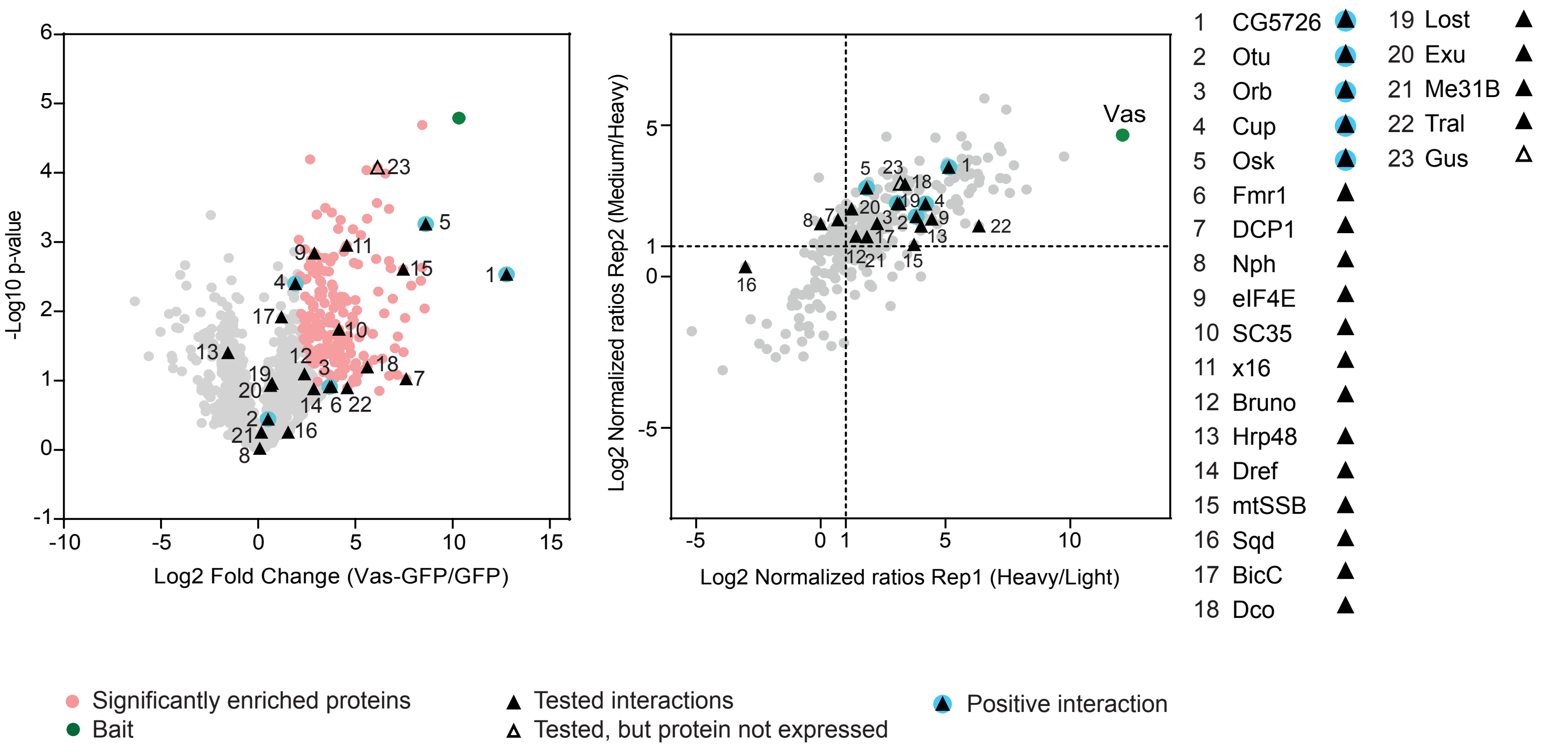

Fig S5. *In vitro* validation screen of selected bait-candidate partners

Volcano plots highlighting all the candidate proteins that were identified in the label-free MS analysis and assayed for interaction with each bait protein, through a co-IP screen in human HEK293 cells. For Hrp48 and Vas, corresponding dimethyl labeling MS information is also shown.

Fig. S6

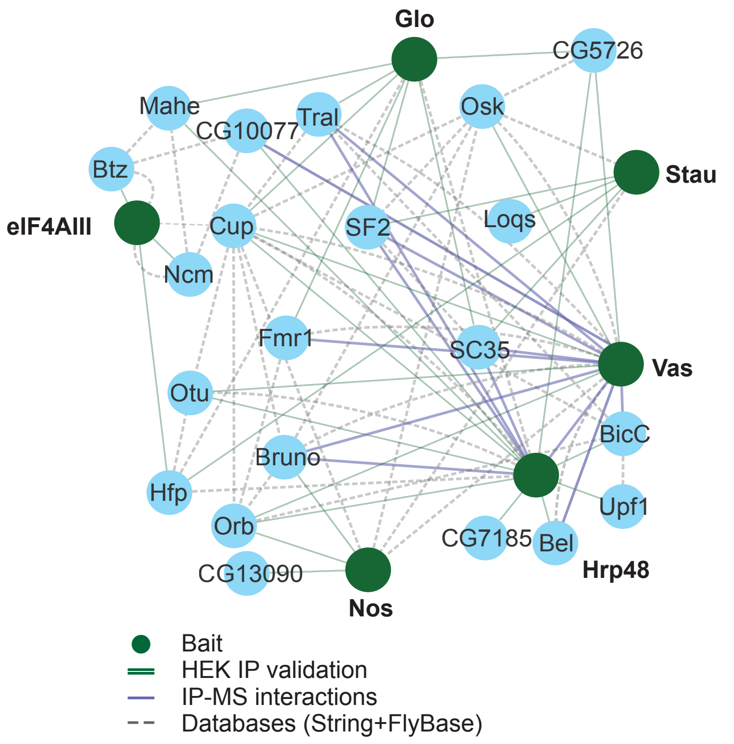

Fig S6. Identification of several novel interactions suggests an extended network underlying *Drosophila* oocyte development

Subnetwork of the protein interactions validated *in vitro*, integrated with the IP-MS data and information from databases.
